# Celiac trunk thrombosis in a patient with antiphospholipid syndrome induced by median arcuate ligament compression: a case presentation and literature review

**DOI:** 10.1007/s00296-023-05448-6

**Published:** 2023-09-27

**Authors:** Paulina Janiak, Żaneta Smoleńska, Monika Skotarczak, Zbigniew Zdrojewski

**Affiliations:** 1 Department of Rheumatology, Hospital in Kościerzyna, Kościerzyna, Poland; 2 Department of Rheumatology, Clinical Immunology, Geriatrics and Internal Medicine, Medical University of Gdańsk, Gdańsk, Poland; 3 Department of Radiology, Medical University of Gdańsk, Gdańsk, Poland

**Keywords:** Median arcuate ligament syndrome, Antiphospholipid syndrome, Thrombosis, Anticoagulant therapy, Abdominal pain

## Abstract

Median arcuate ligament syndrome (MALS) is a rare disorder caused by the compression of the celiac axis by the fibrous structure of the diaphragm called the median arcuate ligament. Patients with MALS are usually undiagnosed unless characteristic symptoms such as nausea and vomiting, postprandial pain, and weight loss are presented. We report a case of a 29-year-old patient diagnosed with MALS and secondary antiphospholipid syndrome (APS) that developed celiac trunk, common hepatic artery and splenic artery thrombosis. There is not enough information on MALS as a trigger of thrombosis in predisposed patients such as those with APS. However, the case gives rise to suspicion and highlights the diagnostic processes, especially for patients with APS presenting postprandial abdominal pain and weight loss. This review likewise aims at the importance of Doppler ultrasonography as a screening tool and computer tomography (CT) or magnetic resonance (MR) both in the angiography variant, especially to diagnose confirmation and underlying treatment options.

## Introduction

Median arcuate ligament syndrome, also called Dunbar syndrome, is a rare condition arising due to the compression of the celiac trunk by the median arcuate ligament (MAL). MAL connects the right and the left diaphragmatic crura at the level of the 12 thoracic vertebra (on average Th11-L1). This fibrous arch forms the anterior edge of the aortic hiatus. In approximately 10–24% of individuals, the MAL is located below Th12/L1 causing some degree of compression and generating abdominal symptoms. Nevertheless, only 1% of this population develops severe stenosis [1, 2]. According to existing data, around 0.002% of the total population experiences MALS [3].

The first clinical case of MALS was reported in 1917 by Lipshutz, followed by angiographically visible compression of the celiac trunk depicted by Harjol and Dunbar in 1963 and 1965, respectively. Median arcuate ligament syndrome is characterized by a triad of symptoms: postprandial pain located in the epigastric area, nausea and vomiting, and weight loss. Patients are usually women with thin body habitus, ages 30–50 [1, 2, 5]. Long-lasting compression of the celiac trunk may lead to intimal growth, a proliferation of elastic fibers in the media and disorganization of the adventitia of vessel wall [4]. There are no standardized diagnostic criteria and MALS is the diagnosis of exclusion. Typical tests that allow differential diagnosis include Doppler ultrasound, CT and MRA of abdominal arteries, and endoscopy. The permitted treatment options for MALS include the laparoscopic release of the median arcuate ligament or open vascular surgery. Most patients do not suffer any symptoms of the disease, and MALS is diagnosed accidentally during the imaging examinations. A significant group of patients diagnosed with compression syndrome does not require treatment [2, 5].

APS is an autoimmune disease, characterized by thrombosis in veins and arteries and the presence of antiphospholipid antibodies (aPl) that includes anti-beta-2 glycoprotein-1, anticardiolipin antibodies, and lupus anticoagulant. The diagnostic criteria (Sapporo criteria) require positivity for aPl, spaced at least 12 weeks apart, and a minimum of one clinical event of vascular thrombosis or obstetrical complications. Based on the etiology, primary APS or secondary with the underlying cause is distinguished. In terms of prevalence, APS ranges between 1 and 2 cases per 100,000 persons per year and is 50–80% higher than the general population. The main treatment strategy includes long-term anticoagulant therapy [6, 7].

## Clinical case

**Fig. 1:**
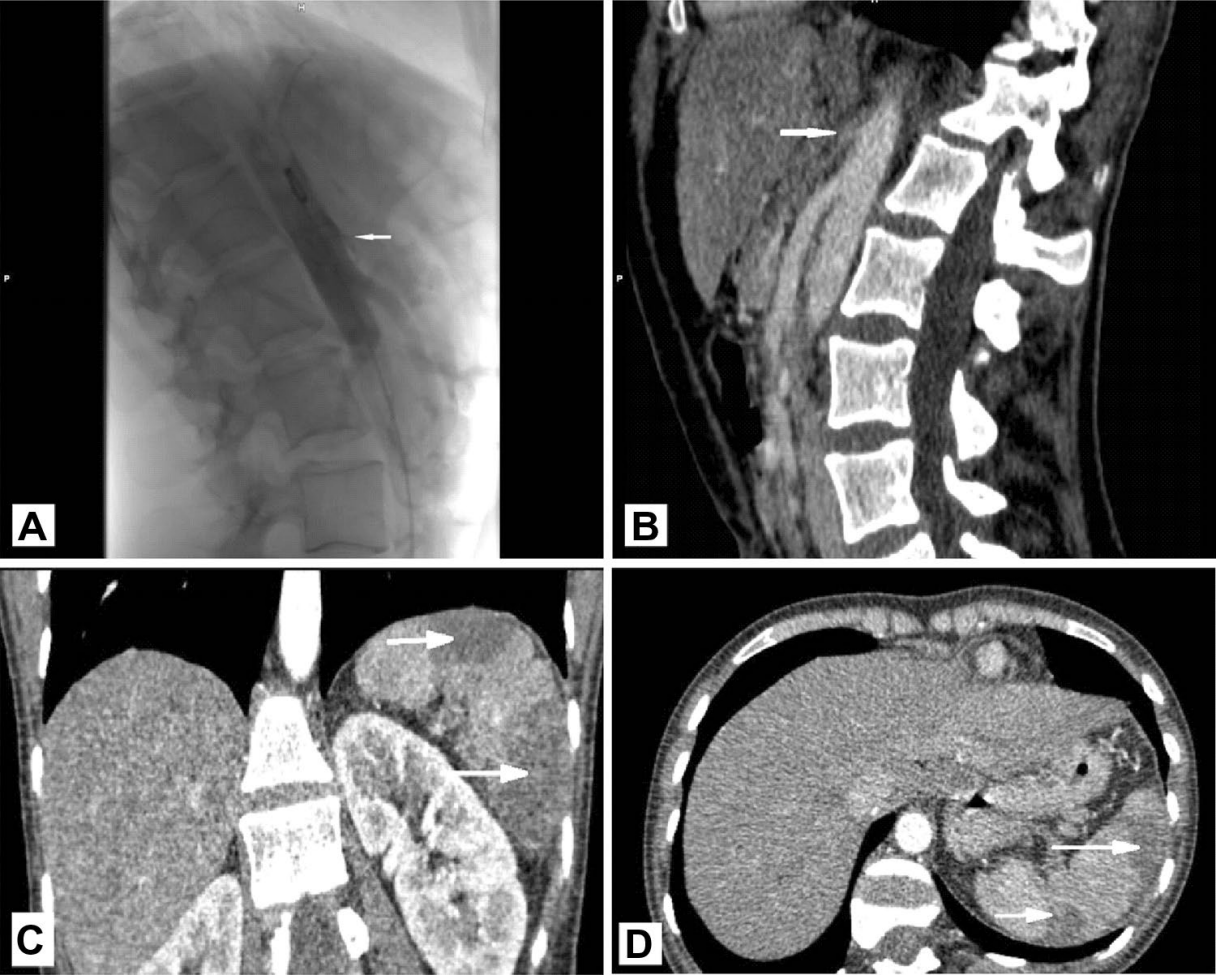
**A** Digital subtraction angiography (DSA) in sagittal plane showing compression of the celiac artery (arrow), **B** contrast enhanced CT image of aorta in sagittal plane showing compression of the celiac artery (arrow), **C** contrast enhanced CT image in sagittal plane showing spleen infarction (arrows), **D** contrast enhanced CT image in transverse plane showing spleen infarction (arrows)

A 29-year old woman was delivered to the emergency department due to abdominal pain, vomiting, nausea, and weight loss. She had a 1-month history of recurrent postprandial abdominal pain. During this time, she lost 10 kg because of lack of appetite and relapsing vomiting. There were no peritoneal irritation signs or significant blood changes. The level of C-reactive protein was slightly high and the number of red blood cells was a bit down. A CT and digital subtraction angiography (DSA) were performed and revealed compression of the celiac artery, spleen infarction (Fig. 1), and thrombosis of the celiac trunk, common hepatic artery, splenic artery with very well-developed collateral circulation. The patient was submitted to a laparotomy where the occluded celiac trunk was visualized and compression by median arcuate ligament was found. The patient has undergone surgery to release the median arcuate ligament; however, vessel revascularization was abandoned. No complications were registered during the postoperative period.

There was no significant family history, she was a non-smoker. The salient point in the patient’s medical record was an unexplained death of a morphologically normal fetus after 12 weeks of the first and the only gestation 2 years before. She also experienced recurrent mild thrombocytopenia, leukopenia but without any therapy, and therefore rheumatology consultation was requested. Deepening the thrombosis diagnosis, it was found that the test for lupus anticoagulant, anticardiolipin antibodies, and anti-β2-glycoprotein-1antibodies was positive and the activity of protein-S was reduced to 47% (reference range 60–130%) (Table 1). Complement C3 component was decreased to 0.44 g/l (reference range 0.9–1.8 g/l) as well as C4 component to 0.06 g/l (reference range 0.1–0.4 g/l). Further immunological investigations were carried out due to the suspected early initial response of autoimmunity. ANAs antibodies titer was determined to be 1:2560 (≥ 1:80 by HEp-2 immunofluorescence). Antibodies against dsDNA and anti-Sm were negative, but she had positive anti-nucleosome antibodies, anti-histone, anti-SSA Ro-60, and anti-Ro-52 antibodies (Table 2). Renal function tests were normal. She had no clinical signs of skin changes, sicca syndrome, and synovitis; however, physical examinations reveal tenderness and pain in multiple small joints of hands and morning stiffness lasting > 30 min. The patient fulfilled relevant 2019 ACR/EULAR Classification Criteria for Systemic Lupus Erythematosus (SLE) [8], and was discharged from the hospital on oral glucocorticoid agent (methylprednisolone 16 mg per day), hydroxychloroquine (0.2 g per day) and had been bridged to warfarin with a therapeutic international normalized ratio (INR) of 2–3. The initial response to the therapy was good. After 12 weeks of treatment, the laboratory investigations showed that the levels of antiphospholipid antibodies were still present but slightly decreased. The patient consequently met the Sapporo criteria for APS, and was diagnosed with secondary APS in the course of systemic lupus erythematosus, coexisting with MALS. She has not developed any other thrombosis involvement during the treatment yet.

**Table 1:** The patient’s thrombosis panel

	The 1st check result	The 2nd check result	The reference range
Anticardiolipin antibodies			
IgG	22.50	15.05	< 10.00 GPL-U/ml
IgM	56.55	32.18	< 7.00 GPL-U/ml
Anti-beta-2-glycoprotein-I antibodies			
IgG	20.61	11.03	< 5.00 GPL-U/ml
IgM	38.22	24.83	< 5.00 GPL-U/ml
Lupus anticoagulant (DRVVT)			
LA1-screening test	78		(31–44) sec
LA2-confirmation test	38		(30–38) sec
L1/L2	202		(0.80–1.20)
Lupus anticoagulant (PTT)			
LA1-correlation test	58		(33–45) sec
LA2-screening test	68		(33–45) sec
Protein C-activity	112		(70–140) %
Protein S-activity	47		(60–130) %
Protein S free-antigen	62		(60–114)%
Antithrombin	113		(83–118) %
Prothrombin time	12		(10–15) sec
International normalized ratio	105		(0.90–1.30)
Activated partial thromboplastin time	38		(26–37) sec

**Table 2:** The patient’s ANA immunoblot assay (Euroimmun)

The type of antibodies	The result	The reference range
Anti-ds/DNA	Negative	Negative
Anti-nucleosome	Positive +	Negative
Anti-histone	Positive + +	Negative
Anti-SSA	Positive + +	Negative
Anti-Ro-52	Positive + +	Negative
Anti-SSB	Negative	Negative
Anti-nRNP	Negative	Negative
Anti-Sm	Negative	Negative
Anti-Mi-2 alpha	Negative	Negative
Anti-Mi-2 beta	Borderline	Negative
Anti-Ku	Borderline	Negative
Anti-centromere A	Borderline	Negative
Anti-centromere B	Negative	Negative
Anti-Sp100	Negative	Negative
Anti-PML	Negative	Negative
Anti-Scl-70	Negative	Negative
Anti Scl-100	Negative	Negative
Anti Scl-75	Positive +	Negative
Anti-RPR11	Negative	Negative
Anti-RPR155	Negative	Negative
Anti-gp210	Negative	Negative
Anti-PCNA	Borderline	Negative
Anti-DFS-70	Negative	Negative

## Search strategy

We performed a search in PubMed, Scopus, and Directory of Open Access Journals up to 2023. A combination of the terms “median arcuate ligament syndrome”, “Dunbar syndrome “, “antiphospholipid syndrome”, and “abdominal thrombotic events” was employed.

## Discussion

Abdominal vessel thrombosis is a rare complication of antiphospholipid syndrome. It usually affects the hepatic vessels, but less frequently the intestinal, spleen, or pancreas vessels. The most common digestive system manifestation of antiphospholipid syndrome is Budd–Chiari syndrome (BCS) that refers to the obstruction of hepatic venous outflow of major hepatic veins [9, 10]. The clinical presentation ranges from an asymptomatic condition to fulminant liver failure but usually shows itself as abdominal pain, ascites, and hepatomegaly [11, 12]. In terms of etiology, primary and secondary types of BCS can be distinguished. Primary BCS is associated with the existence of thrombotic risk factors while secondary is often due to extrinsic compression and tumor infiltration [13–15]. APS can also manifest itself as mesenteric and celiac trunk thrombosis. Rosenthal et al. documented a case of a 39-year old woman diagnosed with SLE and secondary Sjogren’s syndrome, recurrent abdominal pain, and reduced appetite. During the analysis, a very tight stenosis of the celiac artery of  > 90% was noted. She was treated with warfarin with a target INR of 3–4 until a clinical improvement was obtained [16]. Shirish et al. reported that celiac and mesenteric stenosis were most commonly found in atherosclerotic patients [17]. Among all the manifestations of antiphospholipid syndrome, spleen or pancreatitis infarction are distinguished less frequently. Following this, the first case of pancreatic involvement in association with aPL was described by Bird et al., in a patient with severe, intravascular coagulation. The patient had a history of recurrent miscarriage. Consequently, the diagnostic process was conducted, and lupus anticoagulant, anticardiolipin antibodies showed to be positive. In another study of 89 patients with spleen infarction, 39% of them had APS [5, 18].

APS includes a wide range of thrombotic manifestations of the digestive system. However, during a differential diagnosis of abdominal vessel stenosis, it is necessary to exclude certain conditions such as MALS which presumably trigger the thrombotic event, especially with young populations without comorbidities as such atherosclerosis. The carried research showed that MALS may disrupt the hepatic artery hemodynamics and reduce hepatic blood flow velocity in patient with liver transplants [18–20, 28]. Typical tests that allow differential diagnosis include Doppler ultrasound, CT, and MR [1, 4]. Doppler ultrasonography can be an excellent screening tool. Once the diagnosis of MALS is suspected, it can show the blood flow changes of the celiac trunk, its flexion during respiration, and diaphragmatic excursion. [3, 4, 21]. In indication to other condition as BCS, Doppler ultrasonography can point out the narrowing of blood veins [22]. Moreover, in particular cases, we can observe an overlap syndrome where the diagnostic process is challenging. Diagnosing and treating antiphospholipid syndrome concomitant with other conditions may involve close collaboration between the radiologist, surgeon, and rheumatologist. However, the background therapy includes vitamin K antagonists (VKA), with a target of INR 2–3. For those with recurrent thrombosis and a minor risk of bleeding, the insertion of low-dose aspirin (75–100 mg) or the upregulation of the VKA dose with the target of INR 3–4 should be considered [6, 7, 23].There is no substantial evidence for using an alternative way of therapy with NOACs (non-vitamin K oral anticoagulants). Patients treated with NOACs, with regard to those taking VKA, are at a higher risk of recurrence of thromboembolism and bleeding events, especially with triple aPl positivity or arterial thrombosis. The recurrent events mostly occur in the arterial circulation ischemic such as strokes and myocardial infarction [23–27]. Nevertheless, there have been reported cases of APS that NOAC therapy can be beneficial [26]. Cohen et al. published a randomized controlled trial where non-inferior NOACs to VKA were concluded [26]. Based on the above, it is not recommended to implement NOACs as standard anticoagulant therapy; however, it can be considered as a treatment for those who cannot use VKA or are not able to reach the recommended INR level. The proper treatment strategy may be under consideration according to the patient's current condition, although long-term anticoagulant treatment is recommended, especially for patients with secondary APS. Additional therapy with hydroxychloroquine, vitamin D, statins, and sirolimus is also considered for thrombotic APS but that requires further analysis [23–27]. The other aspect that links APS with the gastrointestinal tract is gut microbiota. There are reports that microbiota may have a deep impact on the autoimmunological system and pathogenesis of immune diseases like APS in susceptible patients. In healthy individuals, the microbiota maintains an anti-inflammatory environment as opposed to when homeostasis is impaired and pro-inflammatory interactions predominate. A profound understanding of those interactions could conduct new ways of prevention, diagnosis, and treatment of APS patients [28, 29].

## Conclusion

We presented a case of a patient with a rare phenomenon of nonspecific abdominal manifestations in a course of MALS and APS coexisting with SLE. During the diagnostic process of a patient with recurrent abdominal pain of unclear etiology, particular care should be taken. There are questions arising about whether MALS is an inducting factor for celiac trunk thrombosis and if every patient with APS and celiac trunk thrombosis or its branches require a check for possible MALS coexistence. Changes in arteries that appear due to the compression of the celiac trunk by the median arcuate ligament and the blood flow narrowing may suggest the answer but further investigation is needed. Special attention in the diagnostic process supposed to be paid to radiological examinations, which may lead to breakthroughs in the conducted diagnostics. Therapy should include surgical and pharmacological intervention. Early diagnosis and treatment initiation can be crucial in preventing serious complications and maintaining a patient’s quality of life.

## Data Availability

The data can be requested from the corresponding authors.
